# HNF1α inhibition triggers epithelial-mesenchymal transition in human liver cancer cell lines

**DOI:** 10.1186/1471-2407-11-427

**Published:** 2011-10-05

**Authors:** Laura Pelletier, Sandra Rebouissou, Danijela Vignjevic, Paulette Bioulac-Sage, Jessica Zucman-Rossi

**Affiliations:** 1 Inserm U674 Génomique fonctionnelle des tumeurs solides, Paris, France; 2 Faculté de Médecine, Université Paris Descartes, Paris, France; 3 UMR 144, Institut Curie, Paris, France; 4 Inserm U889, Université Bordeaux 2, Bordeaux, France; 5 Pathlogy department, CHU Bordeaux Hôpital Pellegrin, Bordeaux, France; 6 Oncology department, Hôpital européen Georges Pompidou HEGP, AP-HP, Paris, France

**Keywords:** Hepatocyte Nuclear Factor 1α, hepatocellular adenoma, tumor suppressor gene, benign tumor, siRNA, EMT, TGFβ1

## Abstract

**Background:**

Hepatocyte Nuclear Factor 1α (HNF1α) is an atypical homeodomain-containing transcription factor that transactivates liver-specific genes including albumin, α-1-antitrypsin and α- and β-fibrinogen. Biallelic inactivating mutations of *HNF1A *have been frequently identified in hepatocellular adenomas (HCA), rare benign liver tumors usually developed in women under oral contraceptives, and in rare cases of hepatocellular carcinomas developed in non-cirrhotic liver. HNF1α-mutated HCA (H-HCA) are characterized by a marked steatosis and show activation of glycolysis, lipogenesis, translational machinery and mTOR pathway. We studied the consequences of HNF1α silencing in hepatic cell lines, HepG2 and Hep3B and we reproduced most of the deregulations identified in H-HCA.

**Methods:**

We transfected hepatoma cell lines HepG2 and Hep3B with siRNA targeting HNF1α and obtained a strong inhibition of HNF1α expression. We then looked at the phenotypic changes by microscopy and studied changes in gene expression using qRT-PCR and Western Blot.

**Results:**

Hepatocytes transfected with HNF1α siRNA underwent severe phenotypic changes with loss of cell-cell contacts and development of migration structures. In HNF1α-inhibited cells, hepatocyte and epithelial markers were diminished and mesenchymal markers were over-expressed. This epithelial-mesenchymal transition (EMT) was related to the up regulation of several EMT transcription factors, in particular *SNAIL *and *SLUG*. We also found an overexpression of TGFβ1, an EMT initiator, in both cells transfected with HNF1α siRNA and H-HCA. Moreover, TGFβ1 expression is strongly correlated to HNF1α expression in cell models, suggesting regulation of TGFβ1 expression by HNF1α.

**Conclusion:**

Our results suggest that HNF1α is not only important for hepatocyte differentiation, but has also a role in the maintenance of epithelial phenotype in hepatocytes.

## Background

Hepatocyte Nuclear Factor 1α (HNF1α) is an atypical homeodomain-containing protein that was originally identified as a hepatocyte-specific transcriptional regulator [1]. *In vivo *and *in vitro *models of HNF1α inactivation demonstrated that this transcription factor plays an important role in hepatocyte differentiation and is also crucial for metabolic regulation and liver function [2-5]. Biallelic mutations of *HNF1A *have been identified in about 35% of hepatocellular adenomas (HCA), rare benign liver tumors usually occurring in young women under oral contraceptives, and in rare cases of hepatocellular carcinomas developed in non-cirrhotic liver [6-8]. Recently, HCA has been described as a heterogeneous disease including at least three main subtypes of tumors in which pathological phenotypes are closely related with specific genetic alterations and clinical features [8-12]. HNF1α-mutated HCA (H-HCA) are phenotypically characterized by a marked steatosis [7-9]. In 90% of the cases, H-HCA are sporadic lesions displaying somatic mutations. However, in rare families with an inherited mutation in one allele of *HNF1A*, MODY3 (Maturity Onset Diabetes of the Young type 3) patients are predisposed to develop familial liver adenomatosis that is defined by the presence of more than 10 HCA nodules in the liver [7,13-16]. Thus, *HNF1A *meets the genetic criteria of a tumor suppressor gene [7].

To gain insight into the tumorigenic mechanisms related to HNF1α inactivation, we performed a transcriptomic analysis of H-HCA and identified pathways aberrantly activated in these tumors [17,18]. Previously, we have shown an aberrant activation of glycolysis and lipogenesis, independent of SREBP-1 and CHREBP, that could explain the steatotic phenotype of these tumors. We also identified an activation of mTOR pathway and of the translational machinery, along with an overexpression of several growth factors and oncogenes. We assessed *in vitro *the role of HNF1α in the observed deregulations by inhibiting its endogenous expression in human liver cancer cell lines using small interfering RNA. Here, we analyse the phenotypic consequences of HNF1α inhibition in two hepatic cell lines, HepG2 and Hep3B.

## Methods

### Cell lines and siRNA transfection

HepG2 and Hep3B cells were obtained from the American Type Culture Collection and were cultured in Dulbecco's Modified Eagle Medium with high glucose (Invitrogen) supplemented with 10% fetal calf serum, penicillin 100 IU/ml and streptomycin 100 μg/ml. SiRNA transfections were performed, as decribed previously [17], according to the manufacturer's protocol, in 6 well-plates using the lipofectamine RNAiMax reagent (Invitrogen) with siRNA duplexes targeting *HNF1A *(NM_000545) (Ambion) with sequence: GGUCUUCACCUCAGACACUtt (exon 8-9 3544). Block-iT Alexa Fluor Red Fluorescent Oligo siRNA (Invitrogen) was used as a double-stranded RNA negative control. In most experiments 10 nM of each siRNA was transfected in triplicate, except for dose-effect studies, where several siRNA concentrations were tested (0, 0.01, 0.05, 0.1, 0.2, 0.4, 0.6, 0.8, 1, 5, 10 and 50 nM) in order to obtain different levels of *HNF1A *expression. Cells were prepared for analyses either 3 or 7 days after transfection for HepG2 cells but only after 3 days for Hep3B cells, because HNF1α inhibition could not be maintained until 7 days in this cell line. The absence of cross-reaction of the HNF1α-siRNA duplexes with the *HNF1B *sequence was checked by comparing the expression level of *HNF1A *transcript in cells transfected with siRNA targeting *HNF1A *with the control siRNA-transfected cells.

### Quantitative RT-PCR

Quantitative RT-PCR (qRT-PCR) was performed in duplicate as previously described [19] using pre-designed primers and probe sets from Applied Biosystems (Additional file 1). Ribosomal 18S *(R18S) *was used for the normalization of expression data and the 2^-ΔΔCT ^method was applied. The final results were expressed as the fold differences of target gene expression in HNF1α siRNA compared with control siRNA in cell lines or in tested samples compared with the mean expression value of normal tissues for tumor analysis.

### Western blotting

Western blot analyses were performed as previously described [18] using the primary antibodies specific for E-Cadherin (Cell Signaling Technology, diluted 1:100), HNF1α, Vimentin and N-Cadherin (Santa Cruz Biotechnology, 1:500, 1:200 and 1:200); Polyclonal rabbit anti-actin (Sigma, 1:3000) was used as loading control.

### Immunofluorescence

Cells were grown on slides for 3 or 7 days and fixed with 4% formaldehyde in phosphate-buffered saline (PBS) 1X for 15 min. After washing with PBS, cells were permeabilized with 0.1% triton for 15 mn, washed with PBS, then, cells were incubated with primary antibody overnight. After three washes with PBS, cells were incubated with secondary antibodies for 1 h. The slides were washed, then mounted with VECTASHIELD^® ^Mounting Medium with DAPI (Vector Laboratories). Immunofluorescence images were obtained using a Carl Zeiss Axiophot microscope. All images within one experiment were collected using 63x objective and the same exposure time. The antibodies used were: rabbit anti-E-cadherin (Santa Cruz Biotechnology, 1:100), rabbit anti-N-cadherin (Santa Cruz Biotechnology, 1:100), rabbit anti-Fibronectin (Sigma, 1:100), and the secondary antibodies were anti-mouse and anti-rabbit (GE Healthcare, 1:100, 1:100). Actin was stained by incubating cells for 1 h with Alexa Fluor 488 phalloidin (Molecular Probes, 1:300).

### Migration assays

Boyden chamber migration assays were performed 72 h after transfection using 24-well migration inserts (BD Biosciences). 1,5 × 10^5 ^cells were plated in the upper chamber of the migration insert and they were left to migrate towards medium with serum for 16 h. Cells on the upper side of the insert membrane were removed with a cotton swab, whereas cells that had migrated to the underside of the insert membrane were fixed with 4% formaldehyde in phosphate-buffered saline (PBS) for 15 min. After washing with PBS, cells were permeabilized with 0.1% triton for 15 min, washed with PBS, and stained with hematoxylin. Cells were counted under 300x magnified field, 10 fields were counted for each condition and each condition was done in triplicates.

### Wound-healing assays

HepG2 cells were seeded and transfected in 6-well plates at the density of 5 × 10^5 ^cells per well. After 48 h, a scratch was made through confluent cells with a pipette tip and cells were washed with PBS, and medium without serum was added. Picture&+s were taken just after the scratch was made and at 24, 48 and 72 h afterwards, to monitor cell movements. The experiment was reproduced three times.

### Time lapse microscopy

HepG2 cells transfected with Control or HNF1α siRNA for 3 days in glass-bottom dishes were imaged using 20x objective and Biostation IM at Nikon Imaging Centre at Institut Curie, Paris. Cells were incubated overnight (during 16-18 h) in the Biostation IM and Images were collected every 10 minutes during 16-18 h. The experiment was repeated three times. Data were analysed using MetaMorph image analysis software.

### Patients and samples

Liver tissues were collected in nine French surgery departments from 1992 to 2004. They were immediately frozen in liquid nitrogen and stored at -80°C until used for molecular studies. The whole series of HCA used for the different molecular analyses included 35 H-HCA previously described [8,9,17], and 23 normal livers taken from patients resected with primary liver tumors developed in the absence of cirrhosis. All the patients were recruited in accordance with French law and institutional ethical guidelines. The study was approved by the ethical committee of Hôpital Saint-Louis, Paris, France.

### Statistical analysis

All the values reported are mean ± SD. Statistical analyses were performed using GraphPad Prism version 5 software and significance was determined using either the nonparametric Mann-Whitney test for unpaired data or the two-tailed t-test. Difference was considered significant at *P *< 0.05. In all graphs, *, **, *** indicate difference between groups at *P *< 0.05, 0.01 and 0.001, respectively.

## Results

### HNF1α silencing impairs epithelial phenotype of hepatocyte tumor cells HepG2 and Hep3B

HepG2 et Hep3B cell lines were transfected with siRNA targeting exon 8-9 of *HNF1A *or control siRNA, as previously described [17]. *HNF1A *mRNA inhibition reached 98% and was maximal at 72 h after transfection, as well as the expression of its transactivated gene *FABP1 *[17]. Silencing of HNF1α lasted until 7 days in HepG2, but was not maintained beyond 3 days in Hep3B. Expression of HNF1α homologue, HNF1β, was not diminished by HNF1α siRNA at 24 and 48 h after transfection, assessing that HNF1α siRNA did not target HNF1β mRNA (Additional File 2A).

**Figure 1:**
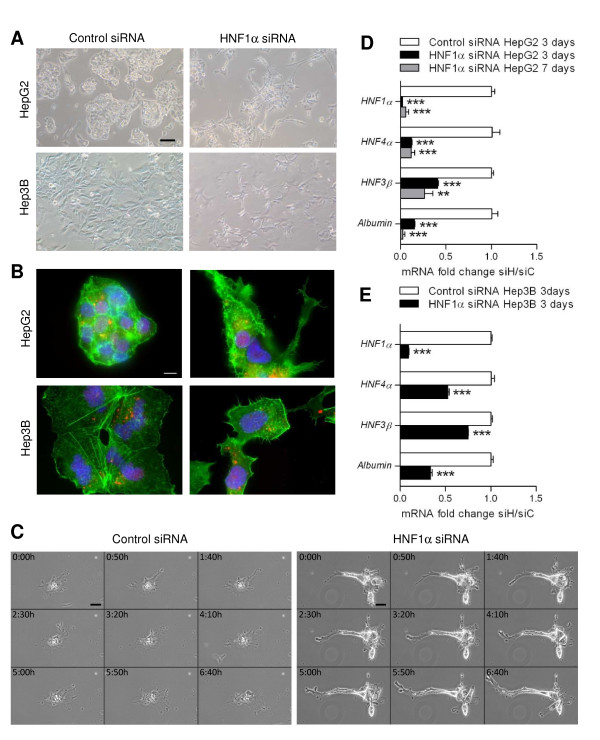
**Loss of epithelial phenotype and development of dynamic structures of migration in HepG2 and Hep3B cells transfected with HNF1α siRNA**. HepG2 and Hep3B cells were transfected with 10 nM of HNF1α siRNA (siH) or with a control siRNA (siC). A: Morphology of transfected HepG2 and Hep3B cells obtained by phase-contrast microscopy with a 10X objective after 72 h. Scale bar 100 μm. B: Actin stained using phalloidin (green) in HepG2 and Hep3B cells transfected with fluorescent control siRNA (red) or co-transfected with fluorescent control and HNF1α siRNA after 72 h. DNA is stained using DAPI (blue). Scale bar 10 μm. C: Time-lapse imaging showing dynamic cytoplasmic protrusions in HepG2 cells transfected with HNF1α siRNA compared to cells transfected with control siRNA, 72 h after transfection. Only 1 image every 50 min is shown here. Scale bar 20 μm. D, E: Expression of transcription factors involved in hepatocyte differentiation and of albumin, a marker of hepatocellular differentiation, in HepG2 (D) and Hep3B (E) cells transfected with HNF1α siRNA after 3 and 7 days for HepG2, compared with cells transfected with control siRNA. mRNA levels were analyzed by qRT-PCR and are expressed as n-fold difference in gene expression of HepG2 cells transfected with HNF1α siRNA (siH) relative to cells transfected with control siRNA (siC) (two-tailed *t*-test).

Cells transfected with HNF1α siRNA had a different phenotype from cells transfected with control siRNA. On phase contrast microscopy, they looked elongated and had lost cell-cell contacts (Figure 1A). This phenotype was maintained until at least 7 days after transfection in HepG2 cells. Phalloidin labelling revealed reorganized actin cytoskeleton with development of actin structures looking like lamelipodia and filopodia in both cell type (Figure 1B). Time-lapse microscopy of HepG2 cells transfected with HNF1α siRNA showed that the cytoplasmic protrusions observed in those cells were dynamic structures protruding from the cell (Figure 1C).

Expression of albumin, a liver-specific gene, and of transcription factors involved in hepatocyte differentiation, assessed by quantitative RT-PCR (qRT-PCR), was diminished 3 days after transfection in both cell type, and was maintained low until at least 7 days after transfection in HepG2 (Figure 1D and 1E). Particularly, HNF4α expression, which has been shown to be regulated by HNF1α [20,21], was decreased early after transfection and this decrease was strongly correlated to HNF1α expression, which was modulated by using several concentrations of siRNA (Additional files 2A and 2B). These results revealed dedifferentiation of cells transfected with HNF1α siRNA.

### Epithelial markers are under expressed and mesenchymal markers are overexpressed in HNF1α-siRNA-transfected cells

**Figure 2:**
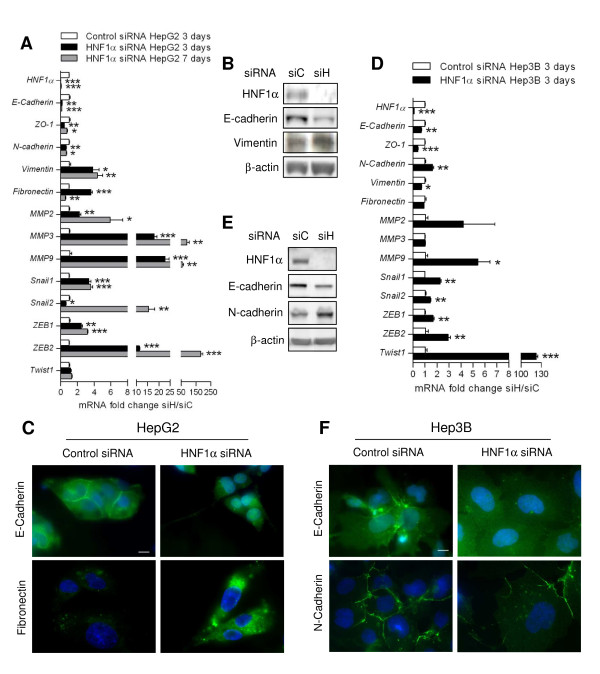
**Expression of EMT markers and transcription factors in HNF1α-inhibited HepG2 and Hep3B**. A,D: mRNA expression levels were compared between HepG2 (A) or Hep3B (D) cells transfected with HNF1α siRNA (siH) and with control siRNA (siC) (two-tailed *t*-test) at 3 and 7 days after transfection. B,E: E-Cadherin, N-Cadherin and Vimentin expression was analyzed using western-blotting after 3 days of transfection with HNF1α siRNA (siH) or Control siRNA (siC) in HepG2 (B) or Hep3B (E). C: Immunofluorescence staining of E-Cadherin, and Fibronectin after 7 days of transfection of Control or HNF1α siRNA in HepG2. Scale bar 10 μm. F: Immunofluorescence staining of E-Cadherin and N-Cadherin after 3 days of transfection of Control or HNF1α siRNA in Hep3B. Scale bar 10 μm.

Epithelial-mesenchymal transition (EMT) is defined by loss of epithelial cell polarity, disappearance of differentiated junctions, reorganization of the cytoskeleton and changes in migration abilities [22-25]. During this process, epithelial markers such as E-cadherin are under expressed and mesenchymal markers are over expressed. In HepG2 cells transfected with HNF1α siRNA, E-cadherin is strongly under expressed at the transcription level as well as at protein level (Figure 2A and 2B). Immunostaining of E-cadherin showed presence at cell-cell junctions in control-siRNA-transfected cells whereas cells transfected with HNF1α siRNA showed no staining at cell borders, suggesting loss of adherens junction in those cells (Figure 2C). Interestingly, the decrease of E-cadherin mRNA was significantly correlated to HNF1α mRNA decrease, when it was modulated using a range of siRNA (Additional file 3). Moreover, zonula occludens-1 (ZO-1), a tight-junction protein, was also under expressed at transcriptional level (Figure 2A). In HNF1α-inhibited HepG2 cells, the mesenchymal markers vimentin and fibronectin were over expressed both at RNA and protein levels (Figure 2A, B and 2C). Several proteins involved in bassement membrane degradation, metalloproteinases (MMP) 2, 3 and 9, were also over expressed in HepG2 cells transfected with HNF1α siRNA (Figure 2A). These characteristics of EMT were observed at 3 days after transfection and were mostly maintained until 7 days.

In Hep3B cell line, epithelial markers were also under expressed and E-cadherin staining was not found at cell border in cells transfected with HNF1α siRNA (Figure 2D, E and 2F). However, the mesenchymal markers over expressed in Hep3B were not the same than in HepG2 cell line. Vimentin and fibronectin remained unchanged whereas N-cadherin was up regulated at RNA and protein levels in Hep3B cells transfected with HNF1α siRNA (Figure 2D and 2E). Overexpression of N-cadherin was not obvious by immunofluorescence analysis, but N-cadherin was mostly found at cell borders and cell-cell contacts were diminished in HNF1α-siRNA-transfected cells (Figure 2F). Finally, metalloproteinase 9 was also significantly over expressed in Hep3B cells transfected with HNF1α siRNA (Figure 2D).

Overall, liver cancer cells transfected with HNF1α siRNA lost expression of epithelial and tight junction markers and over expressed proteins usually expressed in mesenchymal cells, defining an epithelial-mesenchymal transition in those cells.

### Overexpression of transcription factors involved in EMT

Several transcription factors have been involved in the establishment of epithelial-mesenchymal transition, and in particular, in the repression of E-cadherin expression. These transcription factors are usually up regulated during EMT [22-24]. Among these proteins, the Snail family members (Snail1 and Snail2, also known as Snail and Slug) play a key role in EMT. Snail1 was up regulated in HepG2 cells transfected with HNF1α siRNA compared with control siRNA, at 3 days after transfection and until 7 days (Figure 2A). Snail2 was slightly under expressed at 3 days after transfection but it was importantly over expressed at 7 days. The transcription factors of the ZEB family, and particularly ZEB2, were over expressed in HepG2 cells transfected with HNF1α-siRNA at 3 and 7 days after transfection (Figure 2A). Up regulation of all these transcription factors was also observed in Hep3B cells, along with the overexpression of Twist1, another transcription factor involved in EMT (Figure 2D).

### HNF1α silencing enhances migration of HepG2 cell line

**Figure 3:**
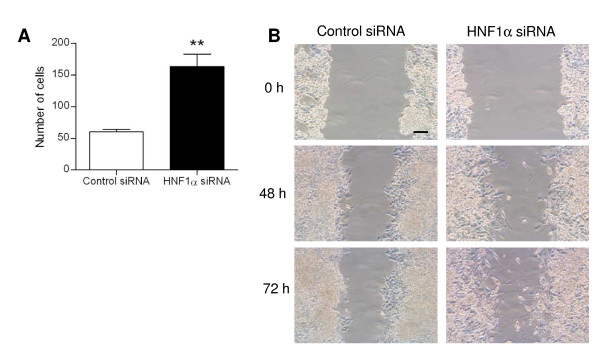
**Migration of HNF1α-inhibited HepG2 cells**. A: Boyden chamber assay. HepG2 cells transfected with control or HNF1α siRNA for 72 h were seeded in insert upper chamber and left to migrate towards medium with serum for 16 h. B: Wound-healing assay. Confluent HepG2 cells transfected with control or HNF1α siRNA for 48 h, were scratched with pipette tips and pictures were taken every day for 3 days. Representative images from three independent experiments are shown. Scale bar 100 μm.

We performed several experiments to assess the ability of migration of HNF1α-inhibited HepG2 cells. First, we put the cells transfected with HNF1α siRNA deprived with serum in a migration insert and let them migrate four 16 h towards medium with serum. More cells were able to migrate when they where transfected with HNF1α siRNA than with control siRNA (Figure 3A).

In a wound healing assay, the scratch caused in cells tranfected with control siRNA did not close completely, even after 72 h (Figure 3B). In HepG2 cells transfected with HNF1α siRNA, the wound did not close completely either but HNF1α-inhibited cells were able to move at the center of the wound unlike control cells (Figure 3B).

Those results showed that HepG2 cells transfected with HNF1α siRNA developed greater migration abilities than control cells.

### TGFβ1 is over expressed in HNF1α-inhibited cells and in HNF1α-mutated hepatocellular adenomas

**Figure 4:**
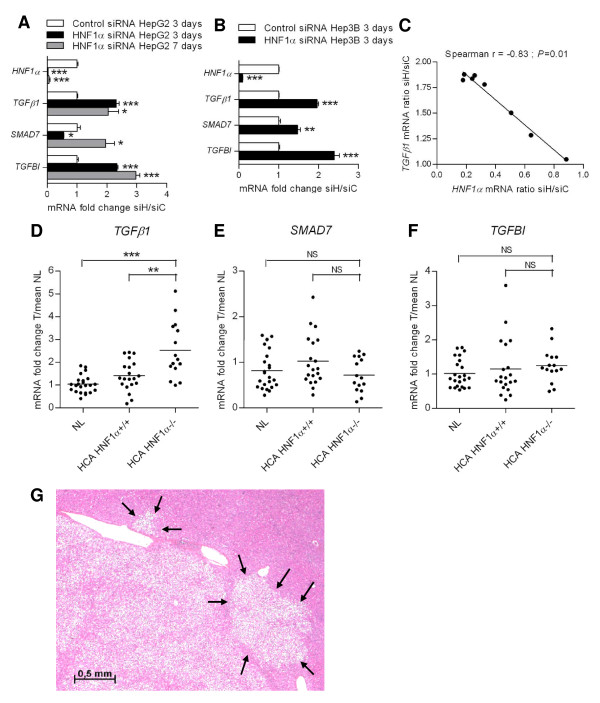
**Overexpression of TGFβ1 in HNF1α-inhibited cells and in HNF1α-mutated HCA**. A,B: mRNA expression levels of *TGFB1 *and its targets *SMAD7 *and *TGFBI *were compared between HepG2 (A) or Hep3B (B) cells transfected with HNF1α siRNA (siH) and with control siRNA (siC) at 3 and 7 days after transfection (two-tailed *t*-test). C: Correlation between expression of HNF1α and TGFβ1 mRNA in HepG2 cells 3 days after transfection were analyzed using a range of siRNA concentrations (0, 0.01, 0.05, 0.1, 0.2, 0.4, 0.6, 0.8 and 1 nM) and significance was assessed by Spearman's rank correlation test. All graphs plot are qRT-PCR results relative to cells transfected with control siRNA. D,E,F: mRNA expression levels of *TGFB1 *(D) and its targets *SMAD7 *(E) and *TGFBI *(F) in HNF1α-mutated HCA (n = 15), HCA not mutated for *HNF1A *(n = 20), and normal liver tissues (NL; n = 23). mRNA levels were analyzed by qRT-PCR and are expressed as n-fold difference in gene expression relative to the mean expression of normal liver tissues (two-tailed Mann-Whitney test). G: Typical aspect of H-HCA featuring marked steatosis and ill-defined borders, with adjacent non-tumoral liver infiltration (indicated by arrows). Hematoxylin-Eosin-Saffron (HES) staining.

Many proteins can trigger epithelial-mesenchymal transition [23,24]. Among them, we found that TGFβ1 was over expressed in HepG2 and Hep3B cells transfected with HNF1α siRNA (Figure 4A and 4B). Moreover, the overexpression of TGFβ1 mRNA was inversely correlated to HNF1α expression in HepG2 cells (Figure 4C). We then studied the transcriptomic expression of two genes that are known to be induced by TGFβ/Smad pathway: *SMAD7*, an inhibitor of TGFβ pathway that is Smad-regulated and is induced by TGFβ in an early response [26,27], and TGFβ-induced (*TGFBI*), an extracellular matrix protein which plays a role in cell-collagen interactions [28]. *SMAD7 *and *TGFBI *were up-regulated at 3 and 7 days after transfection in HepG2 (Figure 4A) and in Hep3B cell lines (Figure 4B). These results suggest that TGFβ pathway is activated in HNF1α-inhibited cells and could participate to the EMT observed in these cells.

Interestingly, we found an overexpression of TGFβ1 in H-HCA compared to normal livers by quantitative RT-PCR (Figure 4D). But we couldn't find any proof of TGFβ pathway activation in these tumors, since neither *SMAD7 *nor *TGFBI *were over expressed, nor any other known TGFβ pathway target genes (Figure 4D, E and data not shown).

## Discussion

HNF1α is a transcription factor involved in hepatocyte differentiation and is important for normal liver function. Here, we show that HNF1α might also be important for maintenance of epithelial phenotype in hepatocytes. Liver cancer cell lines in which HNF1α expression was inhibited by siRNA underwent an epithelial-mesenchymal transition and lost hepatocyte differentiation and epithelial phenotype. Expression of proteins involved in tight and adherens junctions, like ZO-1 and E-cadherin, was diminished, leading to loss of cell-cell contacts and reorganization of cytoskeleton. Cells transfected with HNF1α siRNA also showed an overexpression of mesenchymal markers and of several key transcription factors involved in EMT development, in particular Snail1 and Snail2.

Under-expression of E-cadherin has previously been described in a mouse model of HNF1α inactivation. In this mouse model in which pancreatic β-cell over expressed a dominant-negative mutant of HNF1α, pancreatic islets showed abnormal architecture with, in particular, a reduced expression of E-cadherin [29]. It was then suggested that E-cadherin could be a target of HNF1α. A putative HNF1α binding site was found in intron 2 of human E-cadherin gene and HNF1α acts as an enhancer on the chicken E-cadherin gene but further studies are required to understand the regulation of E-cadherin by HNF1α. Our results showed a strong correlation between E-cadherin and HNF1α expression, supporting the hypothesis of a regulation of E-cadherin expression by HNF1α, whether direct or indirect. HNF1α has also been shown to be a positive regulator of other molecules of cellular junctions, tight junction component claudin-2 [30] and gap junction protein connexin32 [31].

The HNF1 homeoprotein family contains another member apart from HNF1α, HNF1β. HNF1α and HNF1β are highly homologous protein that can recognize the same binding site and form heterodimers [32]. They are both expressed in the polarized epithelium of several tissues (liver, kidney, pancreas and digestive tract), though in a sequential manner, which led to the assumption that they could be involved in epithelial differentiation [33]. In the liver, HNF1β is expressed earlier during development but in adult hepatocytes HNF1α is predominant, whereas HNF1β is weakly expressed [20]. HNF1β inactivation has been linked to EMT in ovarian cancer [34]. Ovarian carcinoma cell lines where HNF1β function was knockdown by siRNA or transfection with a dominant-negative mutant showed reduced E-cadherin expression and underwent epithelial-mesenchymal-like transition, associated with Slug overexpression. HNF1β overexpression lead to down-regulation of Snail and Slug expression. In ovarian tumors, expression of HNF1β was associated with E-cadherin. Altogether, these results support a role of HNF1β in the maintenance of epithelial phenotype. As HNF1α and β have very close activity and can recognize the same genes, HNF1α inactivation in hepatocytes could trigger the same reactions.

Repression of E-cadherin and other epithelial markers by HNF1α could also go through other molecules regulated by HNF1α. In particular, EMT regulators Snail1/2 and ZEB1/2 are able to repress E-cadherin expression through interaction with specific E-boxes of the E-Cadherin promoter [35,36]. Snail1 has recently been shown to be repressed by HNF1α in hepatocytes, through binding of HNF1α to a consensus site in Snail1 promoter [37]. HNF1α can repress Snail1 expression alone or in cooperation with HNF4α, another important regulator of hepatocyte differentiation [37].

Hepatocyte differentiation is achieved through a complex network of cross regulation between transcription factors, especially between HNF1α and HNF4α [20]. There is a regulational hierarchy between those proteins since HNF4α expression precedes that of HNF1α and activates the expression of HNF1α [38]. On the other hand, HNF1α is also capable of activating HNF4α expression, which defines a regulatory loop assuring the expression of HNF1α and HNF4α in hepatocytes [21,39]. Moreover, HNF1α can repress its own expression and the expression of other targets of HNF4α, through interaction with HNF4α [40]. HNF4α has been involved in epithelium formation and it has been shown to regulate the expression of several epithelial markers and components of cell junctions [41,42]. HNF4α has been recently shown to negatively regulate mesenchymal molecules (vimentin, fibronectin and desmin) and EMT master regulator Snail1 [37]. Moreover, HNF4α inactivation induces EMT in embryonic mouse kidneys [43]. Interestingly, HNF1α seems to cooperate with HNF4α to suppress mesenchymal markers expression as well as Snail1 [37]. Since HNF4α was down-regulated in HNF1α-inhibited hepatocytes, the EMT observed in these cells could also go partially through HNF4α inhibition.

Genes involved in cell mobility are also up regulated in HNF1α-inhibited cells, like metalloproteinases, but also PDGFA and B, which have been previously described as over expressed in HNF1α-inactivated tumors and cell lines [17]. PDGF growth factors are involved in angiogenesis but they are also autocrine factors involved in EMT and are necessary for TGFβ-induced migration and tumor progression in hepatocytes [25,44]. Our results show that the EMT induced by HNF1α inhibition is associated with increased cell migration.

To induce EMT, HNF1α could also control directly the expression of growth factors capable of inducing EMT. Among those factors, we showed that TGFβ1 was up-regulated in cells transfected with HNF1α siRNA and that the expression of TGFβ1 was inversely correlated to the expression of HNF1α, suggesting close regulation. Yet it is not clear whether it is this overexpression that trigger the EMT observed in these cells or not. In particular, TGFβ can induce the under expression of HNF4α in rat primary hepatocytes and in immortalized murine hepatocytes [45]. Therefore, HNF4α down regulation in HNF1α-inhibited cells could also be due to TGFβ1 over-expression. Further studies are necessary to understand the role of TGFβ1 overexpression in the development of EMT induced by HNF1α inhibition.

Interestingly, we also found an overexpression of TGFβ1 in HNF1α-mutated HCA, but neither SMAD7 nor TGFBI up-regulation, nor changes in TGFβ-activation markers. Moreover, an analysis of H-HCA transcriptome failed to identify a TGFβ signature in H-HCA, whether early or late, as defined by Courlouarn et al. [26] (data not shown). In particular we didn't identify any change in the expression of EMT markers at the transcriptional level in H-HCA. Neither could we analyze the expression of EMT markers at the borders of these tumors by immunostaining because of the important steatosis observed in H-HCA that makes the staining in tumors highly heterogeneous. However, H-HCA present ill-defined borders, that look like local invasions of the adjacent non tumor liver, which is compatible with EMT (Figure 4G).

The role of TGFβ1 overexpression in these benign tumors remains unclear. TGFβ has a dual effect on tumor development. In early carcinogenesis, TGFβ activation induces cell death and in late carcinogenesis, it is involved in invasion and EMT development [46]. In tumorous cell lines, cells are at a late stage of carcinogenesis and therefore TGFβ is prone to induce EMT. Whereas in benign tumors, we could think that TGFβ overexpression would induce apoptosis but HNF1α-mutated HCA do not show important necrosis and transcriptomic analysis did not reveal important changes in genes involved in apoptosis or cell cycle arrest [17,18]. In the liver, TGFβ has also been involved in hepatic differentiation and fibrosis [47,48]. HNF1α-mutated adenomas are developed in normal livers and do not show fibrosis, so this aspect of TGFβ is irrelevant, but HNF1α and TGFβ are both involved in hepatic differentiation. TGFβ pathway is involved in several steps of liver development, in particular in hepatoblast proliferation and differentiation [48,49]. Weak TGFβ concentrations are needed for hepatoblast differentiation into hepatocytes. As HNF1α is involved in late hepatocyte differentiation, we suggest that HNF1α negative control of TGFβ1 expression could be associated with establishment/maintenance of hepatocyte differentiation and arrest of proliferation.

## Conclusion

In conclusion, our study shows that HNF1α loss can lead to epithelial-mesenchymal transition in liver cancer cell lines, with E-cadherin repression, TGFβ1 overexpression and increased migration abilities. This result suggests that HNF1α could be involved in maintenance of epithelial phenotype in these cell lines and gives new insight in understanding the mechanism related to HNF1α inactivation.

## List of Abbreviations

HNF1α: Hepatocyte nuclear factor 1 alpha; HCA: Hepatocellular Adenoma; H-HCA: HNF1α-mutated Hepatocellular Adenoma; qRT-PCR: Quantitative Reverse Transcription-PCR; EMT: Epithelial-mesenchymal transition; TGFβ: Transforming growth factor beta

## Competing interests

The authors declare that they have no competing interests.

## Authors' contributions

LP, SR, DV, JZR contributed to study concept and design, data acquisition, and data analysis. PBS contributed to data acquisition and data analysis. LP has drafted the manuscript and all other authors critically reviewed the manuscript. All authors gave final approval of the version to be published.

## Pre-publication history

The pre-publication history for this paper can be accessed here:

http://www.biomedcentral.com/1471-2407/11/427/prepub

## Supplementary Material

Additional file 1 TaqMan^® ^pre-designed gene expression assays Click here for file

Additional file 2 **Expression of HNF1β and HNF4α after inhibition of HNF1α expression in HepG2 cells**. **A**: HepG2 cells were transfected independently with siRNA directed against exons 8 and 9 of HNF1α (siH), or with a control siRNA (siC). Inhibition efficiencies were assessed at 0, 1 and 2 days after transfection by measuring the expression level of *HNF1A *and of its transactivated gene (*FABP1*) by qRT-PCR. Expression of homologue HNF1β was measured to assess the specificity of HNF1α siRNA, and HNF4α expression was also measured (two-tailed *t*-test). B: Correlations between expression of HNF1α and HNF4α mRNA were analyzed using a range of siRNA concentrations (0, 0.01, 0.05, 0.1, 0.2, 0.4, 0.6, 0.8, 1, 5, 10 and 50 nM) and significance was assessed by Spearman's rank correlation test. All graphs plot are qRT-PCR results relative to cells transfected with control siRNA. Click here for file

Additional file 3 **E-cadherin expression is correlated to HNF1α expression**. Correlations between expression of *HNF1A *and *CDH1 *were analyzed using a range of siRNA concentrations (0, 0.01, 0.05, 0.1, 0.2, 0.4, 0.6, 0.8, 1, 5, 10 and 50 nM) and significance was assessed by Spearman's rank correlation test. All graphs plot are qRT-PCR results relative to cells transfected with control siRNA. Click here for file
