# Supplementary material for: HNF1α inhibition triggers epithelial-mesenchymal transition in human liver cancer cell lines
Source: BMC Cancer. 2011 Oct 5;11:427. doi: 10.1186/1471-2407-11-427 (PMC3203860; doi:10.1186/1471-2407-11-427)
Supplement: Additional file 1 — TaqMan® pre-designed gene expression assays [file 1471-2407-11-427-S1.DOC]

| **Additional file 1. TaqMan® pre-designed gene expression assays** | | |
| --- | --- | --- |
|  |  |  |
| **Assay ID** | **Gene symbol** | **Gene name** |
| Hs99999901_s1 | 18S | Eukaryotic 18S rRNA |
| Hs00609411_m1 | ALB | Albumin |
| Hs00170423_m1 | CDH1 | cadherin 1, type 1, E-cadherin |
| Hs00169953_m1 | CDH2 | cadherin 2, type 1, N-cadherin |
| Hs00155026_m1 | FABP1 | Fatty acid binding protein 1, liver |
| Hs00170586_m1 | FGB | Fibrinogen, B beta polypeptide |
| Hs00415006_m1 | FN1 | Fibronectin 1 |
| Hs00232764_m1 | FOXA2/HNF3b | Fokhead box A2 / Hepatocyte Nuclear Factor 3 beta |
| Hs00231106_m1 | FOXO1A | forkhead box O1 |
| Hs00167041_m1 | HNF1A | HNF1 homeobox A |
| Hs00230853_m1 | HNF4A | hepatocyte nuclear factor 4, alpha |
| Hs00234422_m1 | MMP2 | matrix metallopeptidase 2 |
| Hs00868308_m1 | MMP3 | matrix metallopeptidase 3 |
| Hs00234579_m1 | MMP9 | matrix metallopeptidase 9 |
| Hs00172885_m1 | NR1H3/LXRa | nuclear receptor subfamily 1, group H, member 3 |
| Hs00231968_m1 | NR1H4/FXR | nuclear receptor subfamily 1, group H, member 4 |
| Hs00234994_m1 | PDGFA | Platelet-derived growth factor alpha polypeptide |
| Hs00234042_m1 | PDGFB | Platelet-derived growth factor beta polypeptide (simian sarcoma viral (v-sis) oncogene homolog) |
| Hs00998193_m1 | SMAD7 | SMAD family member 7 |
| Hs00195591_m1 | SNAI1 | snail homolog 1 |
| Hs00950344_m1 | SNAI2/SLUG | snail homolog 2 |
| Hs00171257_m1 | TGFB1 | transforming growth factor, beta 1 |
| Hs00165908-m1 | TGFBI | transforming growth factor, beta-induced, 68kDa |
| Hs01551861_m1 | TJP1/ZO-1 | Tight junction protein 1 (zona occludens 1) |
| Hs00361186_m1 | TWIST1 | twist homolog 1 |
| Hs00185584_m1 | VIM | vimentin |
| Hs00232783_m1 | ZEB1 | zinc finger E-box binding homeobox 1 |
| Hs00207691_m1 | ZEB2 | zinc finger E-box binding homeobox 2 |
