# Supplementary figures and images for: HNF1α inhibition triggers epithelial-mesenchymal transition in human liver cancer cell lines
Source: BMC Cancer. 2011 Oct 5;11:427. doi: 10.1186/1471-2407-11-427 (PMC3203860; doi:10.1186/1471-2407-11-427)

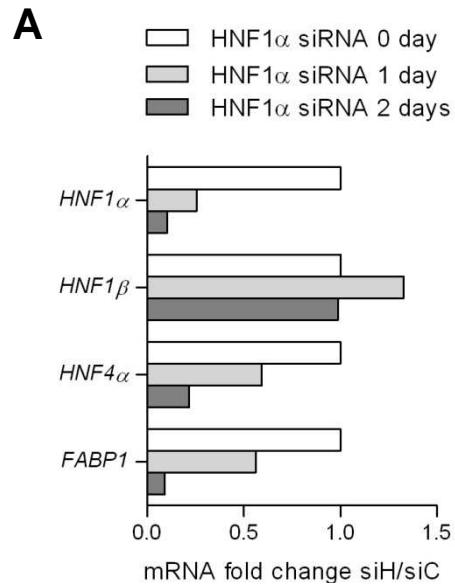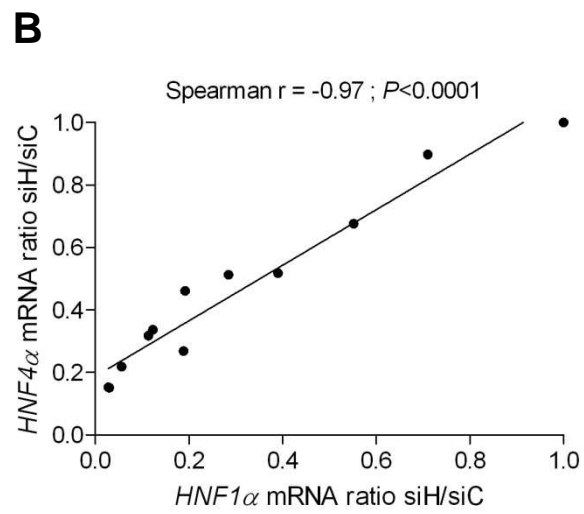

Supplement: Additional file 2 — Expression of HNF1β and HNF4α after inhibition of HNF1α expression in HepG2 cells. A: HepG2 cells were transfected independently with siRNA directed against exons 8 and 9 of HNF1α (siH), or with a control siRNA (siC). Inhibition efficiencies were assessed at 0, 1 and 2 days after transfection by measuring the expression level of HNF1A and of its transactivated gene (FABP1) by qRT-PCR. Expression of homologue HNF1β was measured to assess the specificity of HNF1α siRNA, and HNF4α expression was also measured (two-tailed t-test). B: Correlations between expression of HNF1α and HNF4α mRNA were analyzed using a range of siRNA concentrations (0, 0.01, 0.05, 0.1, 0.2, 0.4, 0.6, 0.8, 1, 5, 10 and 50 nM) and significance was assessed by Spearman's rank correlation test. All graphs plot are qRT-PCR results relative to cells transfected with control siRNA. [file 1471-2407-11-427-S2.PDF]

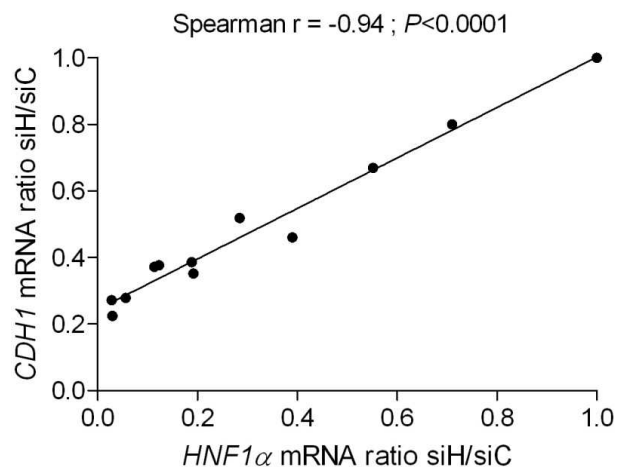

Supplement: Additional file 3 — E-cadherin expression is correlated to HNF1α expression. Correlations between expression of HNF1A and CDH1 were analyzed using a range of siRNA concentrations (0, 0.01, 0.05, 0.1, 0.2, 0.4, 0.6, 0.8, 1, 5, 10 and 50 nM) and significance was assessed by Spearman's rank correlation test. All graphs plot are qRT-PCR results relative to cells transfected with control siRNA. [file 1471-2407-11-427-S3.PDF]
